# A systematic scoping review of ethical issues in mentoring in medical schools

**DOI:** 10.1186/s12909-020-02169-3

**Published:** 2020-07-31

**Authors:** Cheryl Shumin Kow, Yao Hao Teo, Yao Neng Teo, Keith Zi Yuan Chua, Elaine Li Ying Quah, Nur Haidah Binte Ahmad Kamal, Lorraine Hui En Tan, Clarissa Wei Shuen Cheong, Yun Ting Ong, Kuang Teck Tay, Min Chiam, Stephen Mason, Lalit Kumar Radha Krishna

**Affiliations:** 1 Yong Loo Lin School of Medicine, National University of Singapore, NUHS Tower Block, 1E Kent Ridge Road, Level 11, Singapore, 119228 Singapore; 2 Division of Supportive and Palliative Care, National Cancer Centre Singapore, 11 Hospital Dr, Singapore, 169610 Singapore; 3 Division of Cancer Education, National Cancer Centre Singapore, 11 Hospital Dr, Singapore, 169610 Singapore; 4 Palliative Care Institute Liverpool, Cancer Research Centre, University of Liverpool, 200 London Rd, Liverpool, L3 9TA UK; 5 Duke-NUS Medical School, National University of Singapore, 8 College Rd, Singapore, 169857 Singapore; 6 Centre of Biomedical Ethics, National University of Singapore, Blk MD 11, 10 Medical Drive, #02-03, Singapore, 117597 Singapore; 7 PalC, The Palliative Care Centre for Excellence in Research and Education, PalC c/o Dover Park Hospice, 10 Jalan Tan Tock Seng, Singapore, 308436 Singapore

**Keywords:** Mentoring, Ethical issues in mentoring, Mentoring abuse, Mentoring relationships, Mentoring environment, Mentoring in medical schools

## Abstract

**Background:**

Mentoring provides mentees and mentors with holistic support and research opportunities. Yet, the quality of this support has been called into question amidst suggestions that mentoring is prone to bullying and professional lapses. These concerns jeopardise mentoring’s role in medical schools and demand closer scrutiny.

**Methods:**

To better understand prevailing concerns, a novel approach to systematic scoping reviews (SSR) s is proposed to map prevailing ethical issues in mentoring in an accountable and reproducible manner. Ten members of the research team carried out systematic and independent searches of PubMed, Embase, ERIC, ScienceDirect, Scopus, OpenGrey and Mednar databases. The individual researchers employed ‘negotiated consensual validation’ to determine the final list of articles to be analysed. The reviewers worked in three independent teams. One team summarised the included articles. The other teams employed independent thematic and content analysis respectively. The findings of the three approaches were compared. The themes from non-evidence based and grey literature were also compared with themes from research driven data.

**Results:**

Four thousand six titles were reviewed and 51 full text articles were included. Findings from thematic and content analyses were similar and reflected the tabulated summaries. The themes/categories identified were ethical concerns, predisposing factors and possible solutions at the mentor and mentee, mentoring relationship and/or host organisation level. Ethical concerns were found to stem from issues such as power differentials and lack of motivation whilst predisposing factors comprised of the mentor’s lack of experience and personality conflicts. Possible solutions include better program oversight and the fostering of an effective mentoring environment.

**Conclusions:**

This structured SSR found that ethical issues in mentoring occur as a result of inconducive mentoring environments. As such, further studies and systematic reviews of mentoring structures, cultures and remediation must follow so as to guide host organisations in their endeavour to improve mentoring in medical schools.

## Background

Mentoring’s success in nurturing “dynamic, context dependent, goal sensitive, mutually beneficial relationships between an experienced clinician and medical students that is focused upon advancing the development of the mentee” has seen its role in medical school education grow [1]. Yet, mentoring has come under increased scrutiny amidst suggestions that mentoring relationships are poorly assessed and supported [2–4]. These gaps are seen to aggravate power dynamics between mentee and mentor [2] and predispose to grave issues such as bullying and even sexual harrassment [3, 4]. These reports have curtailed mentoring’s role in medical school education, hampering the provision of personalised, longitudinal and holistic support [5–7], career guidance [8–15], and research opportunities [16–22] to medical students.

With mentoring playing a key role in training medical students and with many ethical issues poorly described [2, 23], a systematic scoping review (SSR) is proposed to map prevailing descriptions of ethical issues in mentoring in medical schools to guide a structured and evidence-based response [24–30].

## Methods

An SSR is employed given its wide scope [24]. However, to allow identification of patterns, relationships and disagreements [25] amongst prevailing studies [26], a new approach to SSRs is proposed. Krishna’s novel systematic evidence-based approach (SEBA) seeks to overcome existing concerns over the transparency and reproducibility of SSRs (henceforth SSRs in SEBA). SSRs in SEBA are built upon a constructivist perspective, enabling it to map complex topics from multiple angles [27–29]. Acknowledging the impact of the mentee’s, mentor’s and indeed the host organisation’s (henceforth stakeholders) historical, socio-cultural, ideological and contextual circumstances on their individual perspectives [30], SSRs in SEBA employ a relativist lens. This allows the collation of mentoring experiences from a diverse population of stakeholders in order to provide a holistic picture of ethical issues in mentoring in medical schools [27–29, 31]. To further enhance the trustworthiness of its synthesis, the research team was supported by medical librarians from the Yong Loo Lin School of Medicine (YLLSoM) at the National University of Singapore and the National Cancer Centre Singapore (NCCS). Advice from local educational experts and clinicians at the NCCS, the Palliative Care Institute Liverpool, YLLSoM and Duke-NUS Medical School (henceforth expert team) was also sought. The expert team was consulted at each stage of the SEBA process. The research team also adopted the principles of interpretivist analysis to immerse themselves in the data. A reflexive attitude towards repeated reading and/or analysis of the quantitative data and group discussions enabled the findings to be pieced together in a more meaningful manner.

**Fig. 1:**
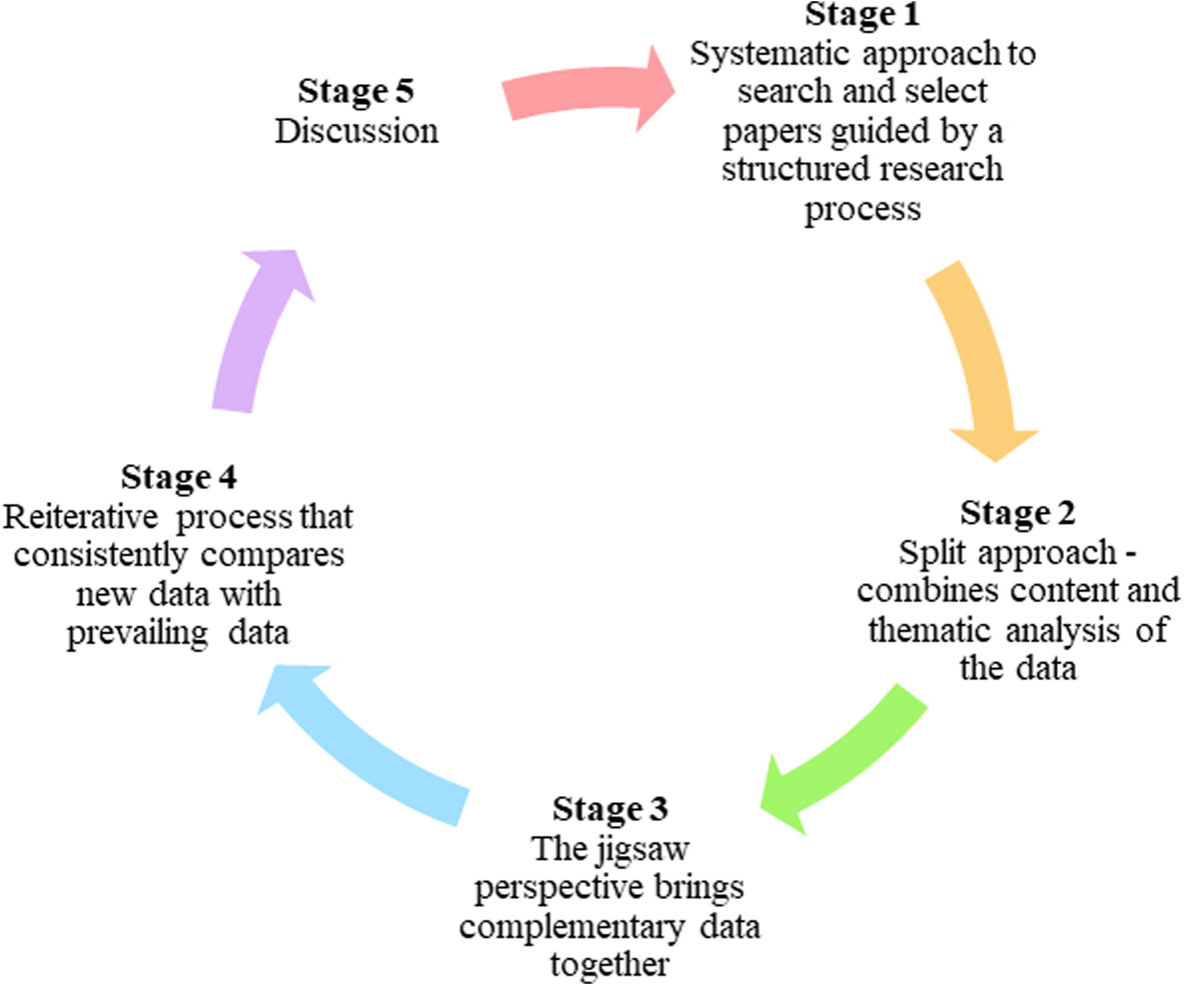
Krishna’s SEBA process

The SEBA process comprises of the following stages: 1) Systematic Approach, 2) Split Approach, 3) Jigsaw Perspective, 4) Reiterative Process and 5) Discussion. This is outlined in Fig. 1 and will be further elaborated on.

### Stage 1: systematic approach

#### Determining title and background of review

Ensuring a systematic approach to the SSR in SEBA synthesis, the research team consulted the expert team to determine the overall goals of the SSR and the population, context and particular mentoring approach to be evaluated.

#### Background of review

Building on findings of two recent systematic scoping reviews [2, 23] of ethical issues in postgraduate and undergraduate mentoring in surgery and medicine, the teams acknowledged that a steeper hierarchy in medical schools see greater power differentials between mentors and mentees as well as the latter’s dependence on the former, thus differentiating ethical issues in medical schools from those affecting doctors.

#### Identifying research question

Guided by the PCC (population, concept and context) elements of the inclusion criteria [32], the primary research question was determined to be “what is known of ethical issues in mentoring within medical schools?” The secondary research questions were determined to be “what factors precipitate ethical issues in mentoring?” and “what solutions have been offered to address ethical issues in mentoring?”

#### Inclusion and exclusion criteria

The inclusion and exclusion criteria are elaborated on below (Table 1).

**Table 1:** PICOS, inclusion criteria and exclusion criteria applied to literature search

PICOS	Inclusion criteria	Exclusion criteria
**Population**	Medical students	Allied health specialties such as dietetics, nursing, psychology, chiropractic, midwifery, social work
**Intervention**	Mentoring of medical students by clinicians Medical specialties related to internal medicine, family medicine, academic medicine and surgical specialties	Non-medical specialties such as clinical and translational science, veterinary, dentistry Non-surgical specialties including anesthesiology and obstetrics and gynecology Role modelling, coaching, supervising and advising
**Comparison**	None	
**Outcome**	Attitude of health personnel Interprofessional relations Ethical behaviour Professionalism Problems/barriers of mentoring	
**Study design**	All study designs are included - Descriptive papers - Qualitative, quantitative, and mixed study methods - Perspectives, opinions, commentary pieces and editorials	

#### Types of population, concept and context

The focus was confined to ethical issues in mentoring in undergraduate and postgraduate medical schools.

#### Searching

Ten members of the research team carried out independent searches of five bibliographic databases (PubMed, Embase, ERIC, ScienceDirect and Scopus) and two grey literature databases (OpenGrey and Mednar) between 5th March 2020 to 7th March 2020 for articles published between 1st January 2000 to 31st December 2019. The PubMed search strategy may be found in Additional file 1.

#### Extracting and charting

**Fig. 2:**
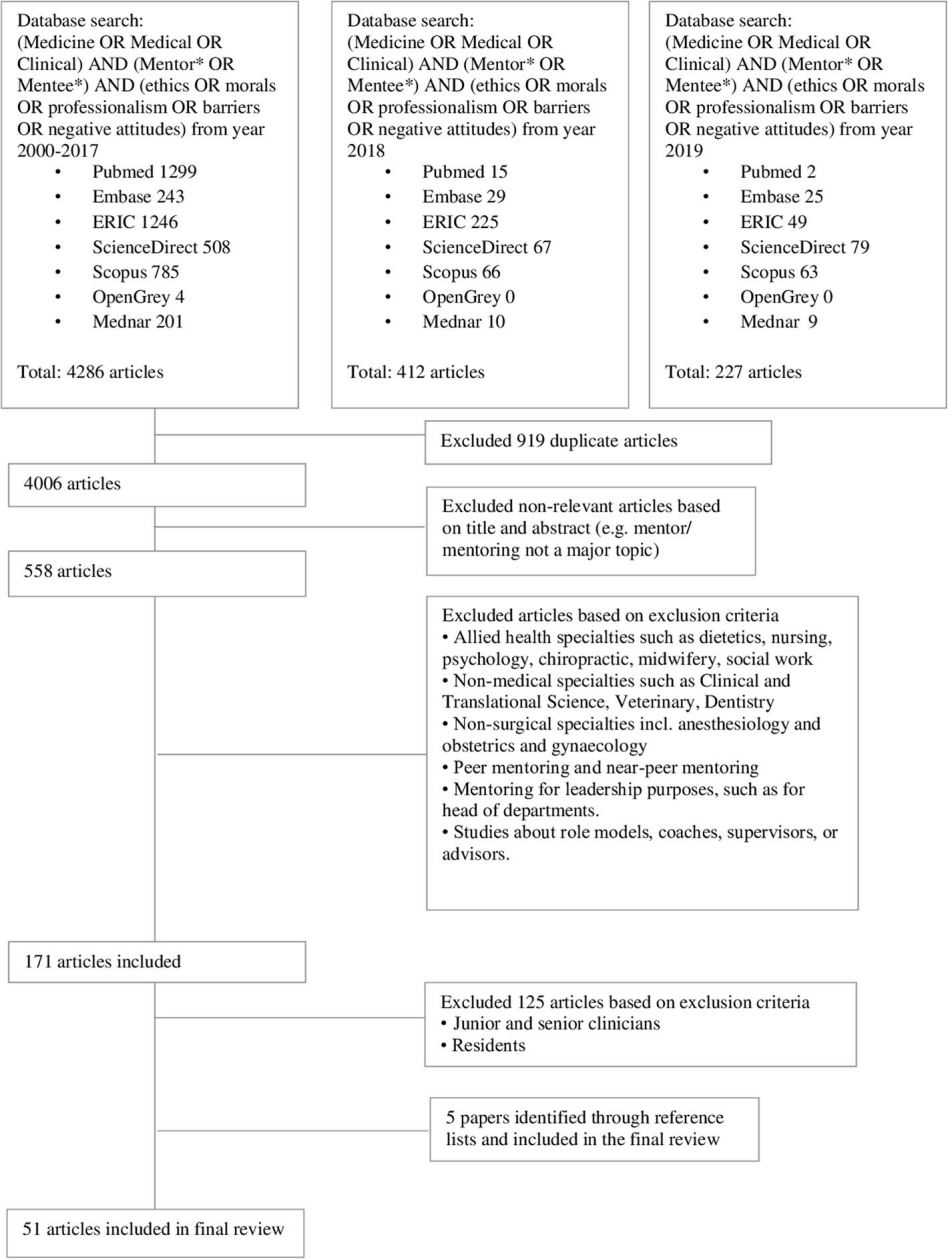
PRISMA Flow chart

Ten members of the research team independently reviewed all identified article titles and abstracts, created individual lists of titles to be included and discussed them online where ‘negotiated consensual validation’ [33] was employed to determine the final list of titles to be reviewed. The team then independently reviewed these titles, compared their individual lists of articles to be included and employed ‘negotiated consensual validation’ once more to achieve consensus on the final list of articles to be analysed. This process is outlined in Fig. 2 and the final list of included articles may be found in Additional file 2.

### Stage 2: split approach

Three teams comprising of three researchers simultaneously reviewed the 51 included full text articles. The first team independently summarised and tabulated the articles in keeping with Wong and Greenhalgh’s RAMESES publication standards: meta-narrative reviews [34] and Popay et al.’s “Guidance on the conduct of narrative synthesis in systematic reviews” [30]. The final table was then agreed upon. It ensured that key discussion points and contradictory views within the included articles were not lost.

Concurrently, each member of the second team analysed the 51 included articles using Braun and Clarke’s approach to thematic analysis [35] whilst the third team used Hsieh and Shannon’s approach to directed content analysis [36]. A comparison of findings from the thematic analysis, directed content analysis and the tabulated summaries is a hallmark of the ‘Split Approach’ used to enhance their reliability.

## Results

### Themes identified using thematic analysis

The themes identified were ethical concerns and solutions at the level of the mentee, mentor, mentoring relationship and host organisation.

### Categories identified using directed content analysis

Deductive category application [37] was employed in tandem with the categories drawn from Cheong et al.’s systematic scoping review of ethical issues in mentoring in internal medicine, family medicine and academic medicine [23] and Lee et al.’s focus on surgery [2] to discern if any new categories could be identified. No new themes were identified beyond those ascertained from Braun and Clarke’s approach to thematic analysis.

### Comparisons between tabulated summaries, thematic analysis and directed content analysis

There was general consensus within the research team and expert team that the findings reflected the positions of the included articles. The combined findings of the Split Approach are presented in Tables 2 and 3.

**Table 2:** Ethical concerns at the mentee, mentor, mentoring relationship and host organisation level

Ethical Concerns at the Level of	Elaboration	References
Mentee	• Lack of Motivation	[38, 39]
	• Poor Collaboration	[40, 41]
Mentor	• Lapses in Professionalism	[42, 43]
	• Failure to Acknowledge Mentee’s Contributions	[38]
	• Lack of Motivation and Commitment	[38–40, 43–45]
	• Poor Collaboration	[46]
Mentoring Relationship	• Power Differentials	[47, 48]
	• Poor Communication	[49]
	• Stifling of Mentee Development • and Competition with Mentee	[38, 45, 50]
	• Gender Based Obstacles	[38, 40, 46, 48, 51–54]
	• Cultural Differences such as with Minority Groups	[38, 48]
	• Personality Conflict	[33, 45, 46, 50, 55, 56]
	• Misalignment of Goals	[55]
Host Organisation	• Poor Recruitment and Recognition	[33, 50, 57, 58]
	• Lack of Clear Guidelines	[59, 60]
	• Poor Oversight and Lack of Assessment	[44, 53]
	• Lack of Administrative Support such as Provision of Protected Time	[33, 40, 43, 44]

**Table 3:** Solutions at the mentee, mentor, mentoring relationship and host organisation level

Solutions to Ethical Concerns at the Level of	Elaboration	References
Mentee and Mentor	• Provision of Appropriate Training	[45, 46, 50, 61–63]
	• Provision of Protected Time	[44, 61–63]
	• Recognition of Mentors	[33, 39, 50, 62]
	• Provision of Financial Support	[33, 50, 62]
	• Facilitation of Group Mentoring with Near-Peer and Senior Peer Mentors to Better Fulfil Mentee’s Needs	[48, 54, 62, 64–66]
	• Alignment of Expectations to Better Meet Respective Responsibilities	[45, 50, 55, 61]
	• Setting of a Clear Code of Conduct for Mentees and Mentors to Better Meet Respective Responsibilities	[50, 55]
Mentoring Relationship	• Provision of Appropriate Training	[38, 48–50, 62, 67]
	• Clear Guidelines and Codes of Conduct to Ensure Clear Boundaries and Alignment of Expectations	[49, 50, 56, 62, 67]
	• Improved Assessment of Mentoring Relationships	[33, 60, 62, 67–69]
Host Organisation	• Facilitation of Mentee-Mentor Matching Process	[40, 50, 58, 61, 62]
	• Incorporation of Mentoring into Curricula	[40, 44]
	• Provision of Administrative and Financial Support	[60–62]
	• Oversight to Identify Breaches in Codes of Conduct	[50, 61]

### Stage 3: jigsaw perspective

The jigsaw perspective brings data from complementary pieces of the mentoring process such as aspects of the mentoring relationship together to create a cohesive picture of ethical issues in mentoring.

### Stage 4: reiterative process

Whilst there was consensus on the content and arrangement of the themes/categories identified, the expert team raised concerns that data from grey literature, which is neither quality assessed nor necessarily evidence-based, could ‘slant’ the direction of the narrative synthesis. As a result of these concerns, the research team thematically analysed the data from grey literature and non-research-based pieces drawn from the five bibliographic databases. These included letters, opinion and perspective pieces, commentaries and editorials.

Comparisons of themes from grey literature and non-research-based pieces with peer-reviewed evidence-based data revealed that the former two additionally focused on predisposing factors to ethical issues involving mentees, mentors and their mentoring relationships (Table 4).

**Table 4:** Predisposing factors to ethical issues found in grey literature and non-research-based pieces

Level of Mentorship Process	Predisposing Factors Leading to Ethical Issues
Individual Mentor/Mentee	• Mentor’s Lack of Experience [50, 51, 56]
Mentoring Relationship	• Personality Conflicts [46, 50, 55, 56] • Mentee’s Over-dependence on Mentor [51] • Minimal Quality Interaction with Mentor [56] • Generational Differences between Mentee and Mentor [55]

### Stage 5: discussion

It comes as no surprise that ethical concerns surrounding mentoring issues in medical schools remain poorly understood and categorised. In curating and analysing the data, this SSR in SEBA found great diversity in what constitutes ethical issues in mentoring. This ranges from ineffective mentoring support and the misappropriation of the mentee’s work [33, 50–52] to suggestions of bullying and potential sexual misconduct [3, 70, 71]. However, despite this diversity, the SSR in SEBA identified patterns and relationships amongst prevailing studies [25, 26]. Indeed, there is consensus amongst the research and expert teams that ethical concerns revolve around and are precipitated by stakeholders and their mentoring relationships. This enabled us to address our research questions:

#### What is known of ethical issues in mentoring within medical schools?

There appears to be a connection between relatively mild or innocuous issues and severe breaches of professional, ethical and clinical codes of conduct. Without timely and effective interventions, innocuous ethical issues may lead to more serious violations in mentoring practice. Poor access to mentoring support [61] may exacerbate power differentials [33, 50, 55, 64] whilst minor lapses in professionalism [42] and a lack of respect for personal and professional boundaries [38, 47, 68] may precipitate bullying, racism, sexism, misappropriation of the mentee’s work and potential sexual abuse [32, 49–51]. Just as concerning is the stiff competition between mentees and mentors for educational and financial resources, with mentors prioritising their own interests over those of their mentees [33, 38, 45, 61, 72].

#### What factors precipitate these ethical issues?

Upon closer analysis of the data, predisposing factors to breaches in ethical mentoring practice may be categorised at the level of the (i) individual mentee and mentor, (ii) mentoring relationship as well as the (iii) host organisation.

At the mentee and mentor level, ethical concerns stem from poor mentee and mentor recruitment and training [33, 40, 44, 47, 50, 52, 58, 62, 73], matching [74], alignment of expectations [40, 44] and oversight and support for the evolving mentoring relationship [57]. These findings highlight three issues. Firstly, mentees and mentors are fundamentally unaware of what is expected of them. Secondly, both groups are not adequately trained to meet their mentoring roles and responsibilities. Thirdly, poor support gives way to communication breakdowns and heightened power dynamics [33, 50, 55, 64], rendering both groups less likely to invest in and sustain their mentoring relationship [39, 43, 49, 63, 75]. Inevitable compromises to the mentoring relationship give rise to significant ethical issues and this failed relationship results in a waste of precious mentoring resources and a missed opportunity for other potential mentees to access the program [39].

At the matching stage, a lack of choice in mentors and poor consideration of mentee preferences for the mentor’s gender, cultural background, experience, interests, personality and working style may have a detrimental effect on the suitability of the mentoring approach and the quality of their interactions [3, 33, 38, 40, 43, 44, 51–55, 63, 66, 76–78]. Poor quality interactions may result from personality conflicts, time pressures, a lack of privacy and difficulties in balancing mentoring goals, outcomes, individual interests and institutional objectives [33, 38–41, 43–46, 50, 52, 55–58, 60, 62, 63, 65, 67, 75, 79]. Compromises to the development of trusting and enduring mentoring relationships result in poor mentoring outcomes, experiences and culminate in the program’s overall failure to meet their participants’ needs.

At the level of the host organisation, the primary source of ethical issues in mentoring revolves around poor structuring of the mentoring program which includes the lack of clearly established goals, timelines, project outcomes, mentoring approaches, roles, responsibilities, codes of conduct and expectations upon mentee, mentor and the host organisation at recruitment, training and matching processes. These issues, if insufficiently addressed or supported by the institution, will lead to mentoring failures.

#### What solutions have been offered to address these concerns?

On deeper consideration of the findings, it could be surmised that the solutions proffered ultimately pivot on the provision of a conducive mentoring environment. To this end, Hee et al. note that an effective mentoring environment comprises of both an efficacious mentoring structure and mentoring culture [80]. The mentoring structure ensures that the various stages of the mentoring process are consistently designed, supported and assessed. This allows for lapses at any stage to be addressed early, minimising the potential snowballing of minor breaches into severe ethical violations. The mentoring culture, in turn, nurtures personal, professional, research, academic, clinical values and behaviours in ways that are consistent with regnant practices and codes of conduct delineated by the respective healthcare and educational systems. It is the mentoring culture that guides mentees, mentors and the host organisation as they navigate through the mentoring process. It informs their decision making within the mentoring relationship and the larger mentoring program.

With the lack of in-depth discussion in prevailing literature on pertinent characteristics of unethical mentoring practice, it is clear that there are no ‘magic bullets’. However, comparing the findings of this SSR in SEBA with existing reviews on mentoring in medicine and surgery [2, 23], it would appear that mentoring in medical schools will benefit from a formal, structured mentoring program overseen by the host organisation. The data suggest that clear guidelines with regards to recruitment, training, matching and the firm delineation of mentee and mentor roles and responsibilities should be established. In addition, Codes of Practice and acceptable parameters should be drawn up and cogently conveyed to all stakeholders. Rigorous, holistic and longitudinal assessments will help to ensure that ethical breaches or violations do not slip through the cracks. In turn, timely, specific, personalised and appropriate support by host organisations will allow mentees to be better protected from many of the ethical issues raised. Suggestions of concrete actions to be taken by the host organisation are outlined in Table 5.

**Table 5:** Suggestions of actions to be taken by host organisation

Process of mentorship	Concrete actions to be taken by host organisation	Purpose of action
Recruitment stage	• Establish clear goals of the mentoring process • Set out the mentoring approach to be used, and support mechanism and assessment programs that will be applied • Ensure that clear recruitment and entrance requirements are established • Expand pool of potential mentors for a suitable mentor-mentee ratio and to increase the intake of female mentors and mentors from different ethnic groups and cultures [33, 73]	• To avail mentoring opportunities to students [57] • To help recruit mentees and mentors with appropriate goals and desired characteristics • To help align expectations of the mentoring program [1, 61]
Matching and Training process	• Aim to provide a personalised and complementary mentorship [58, 61] • Provide mentees and mentors with a chance to meet and discuss their individual goals, values, timelines, interests, working styles and availabilities in pre-mentoring meetings before the mentoring relationship begins [61] • Provide mentees and mentors with a ‘two week’ trial period to confirm that they intend to proceed with the mentoring relationship [61] • Training sessions to be mandatory and longitudinal for all mentors and mentees [33, 61] • Establish a Code of Practice – this includes codes of conduct, standards of practice, professional codes of practice and institutional expectations and guidelines [3, 81]	• To help mentees find the right mentoring relationship and to inform them of boundaries to abide by [41, 73] • To convey expectations of the mentoring program and to ensure awareness of the roles and responsibilities of the mentor [23] • To ensure that mentors and mentees gain skills required to maintain a healthy mentoring relationship • To offer a chance to gather feedback on mentoring progress, support and the program as a whole [61] • To ensure that mentors and mentees are supported throughout their mentoring journey [23] • To provide clear guidance for mentors and mentees on Codes of Practice, how it will be policed and ramifications of minor breaches or severe violations
Mentoring process	• Schedule regular meetings between all stakeholders [45, 55] • Provide effective communication platforms [54] • Encourage open and frank discussions, exchange of ideas and feedback which would the facilitate the building of successful mentoring relationships [43] • Provide recognition of contributions of mentors and mentees such as through certification or awards [55, 72] • Access to longitudinal administrative and financial support	• To provide a nurturing mentoring environment • To ensure that there are opportunities for feedback, support and honest conversations • To ensure a consistent mentoring approach • To ensure that mentors are aware of ways they may benefit personally, professionally and be formally recognised for their contributions so that they may remain incentivized and committed to the mentoring program and their objectives [55, 72] • To provide encouragement and support for mentorship; ensuring balance between individual’s goals and interests with pressures and expectations
Evaluation of mentorship	• Set up a robust and structured assessment program [51] through the selection of assessment methods and domains that are context-, mentee- and mentor-specific. Assessment may also be anonymous and should be confidential and non-threatening to encourage honesty from mentees and mentors • Longitudinal evaluation of the mentorship through feedback mechanisms in assessing outcomes of mentorship [60] • If issues surface, introduce remediation courses for mentors and mentees so as to repair the mentoring relationship and to prevent further ethical breaches or violations [75]	• To ensure that the mentoring approach employed is well articulated and adhered to • To address ethical issues in mentorship such as lapses in professionalism, conduct, collaborative efforts, motivation and widening power differentials in a timely manner • To address issues in the mentor-mentee relationship, such as misalignment of expectations, poor recruitment and training, inadequate communication skills, insight, support and training

## Limitations

Efforts to enhance the reproducibility and transparency of the SSR in SEBA unfortunately appear limited. Whilst the databases used were identified by the expert team, critical papers may still have been omitted despite concerted efforts to employ independent selection processes. Similarly, whilst use of the Split Approach allowed for triangulation and greater transparency of the SSR in SEBA, inherent human biases may have impacted the data analyses. Although use of thematic analysis to review the impact of grey literature also improved the transparency of the discussion, the inclusion of themes derived from grey literature may have skewed the results by providing opinion-based views with a ‘veneer of respectability’ despite a lack of evidence to support them. This raises the question as to whether grey literature should be accorded the same weight as published literature.

## Conclusion

In answering its primary and secondary research questions, this SSR in SEBA provides a comprehensive, accountable, reproducible and evidence-based perspective of ethical issues in mentoring in undergraduate and postgraduate medical schools. This SSR in SEBA also identifies the pivotal role of the host organisation in identifying and addressing these concerns.

Further studies on mentoring structures and mentoring cultures are necessary to further guide efforts on how a more gracious and altruistic mentoring environment could be facilitated. Better prevention and remediation of ethical breaches and violations would also certainly help to reinstate mentoring’s role in medical schools. It is only when these concerns are addressed head-on that all stakeholders will be able to truly reap the benefits of mentoring.

## Supplementary information

**Additional file 1.** Search Strategy, which shows Search Strategy employed for PubMed.

**Additional file 2.** Summary of Included Articles, which shows Summary of Included Articles.

**Additional file 3.** PRISMA Checklist.doc, PRISMA Checklist, which shows the PRISMA checklist for this review.

## Data Availability

All data generated or analysed during this study are included in this published article and its supplementary information files.

## Abbreviations

SSR: Systematic Scoping Review
SEBA: Systematic Evidence-Based Approach
YLLSoM: Yong Loo Lin School of Medicine
NCCS: National Cancer Centre Singapore
PCC: Population Concept Context
PICOS: Population Intervention Comparison Outcomes Study Design
