# Supplementary material for: A systematic scoping review of ethical issues in mentoring in medical schools
Source: BMC Med Educ. 2020 Jul 31;20:246. doi: 10.1186/s12909-020-02169-3 (PMC7395401; doi:10.1186/s12909-020-02169-3)
Supplement: Supplementary file 1 — Additional file 1. Search Strategy, which shows Search Strategy employed for PubMed. [file 12909_2020_2169_MOESM1_ESM.doc]

*Additional file 1 – Search Strategy*

| **Database** | **Search Terms** |
| --- | --- |
| Pubmed | ((((((("Education, Medical, Graduate"[Mesh] OR "Education, Medical, Undergraduate"[Mesh] OR "Internship and Residency"[Mesh] OR "Pediatrics/education"[Mesh] OR "Reproductive Medicine/education"[Mesh] OR "Specialties, Surgical/education"[Mesh] OR "Psychiatry/education"[Mesh] OR "Physician's Role"[Mesh] OR "Surgical Procedures, Operative/education"[Mesh] OR "Medicine/education"[Mesh] OR medicine[tiab] OR medical [tiab] OR clinical[tiab]) AND (undergraduate[tiab] OR postgraduate[tiab] OR training[tiab] OR trainings[tiab] OR trainee[tiab] OR trainees[tiab] OR trainer[tiab] OR trainers[tiab] OR student[tiab] OR students[tiab] OR intern[tiab] OR interns[tiab] OR internship[tiab] OR internships[tiab] OR interning[tiab] OR resident[tiab] OR residents[tiab] OR residency[tiab] OR residencies[tiab] OR physician [tiab] OR physicians [tiab] OR clinician [tiab] OR clinicians [tiab] OR professor [tiab] OR professors [tiab] OR faculty [tiab] OR teacher [tiab] OR teachers [tiab] OR teaching[tiab] OR teachings[tiab] OR education[tiab] OR educations[tiab] OR competence[tiab] OR competency[tiab])) AND ( "2000/01/01"[PDat] : "2017/12/31"[PDat] ))) AND ("Mentoring"[Mesh] OR "Mentors"[Mesh] OR Mentor[tiab] OR mentors[tiab] OR mentorship[tiab] OR mentorships[tiab] OR mentee[tiab] OR mentees[tiab] OR mentoring[tiab] OR mentor-mentee[tiab] OR mentee-mentor[tiab] OR mentored[tiab])) AND ("Attitude of Health Personnel"[Mesh] OR "Interprofessional Relations"[Mesh] OR "Workplace/psychology"[Mesh] OR "Ethics"[Mesh] OR "Professionalism"[Mesh] OR "Morals"[Mesh]) |
