# Supplementary material for: A systematic scoping review of ethical issues in mentoring in medical schools
Source: BMC Med Educ. 2020 Jul 31;20:246. doi: 10.1186/s12909-020-02169-3 (PMC7395401; doi:10.1186/s12909-020-02169-3)
Supplement: Supplementary file 2 — Additional file 2. Summary of Included Articles, which shows Summary of Included Articles. [file 12909_2020_2169_MOESM2_ESM.docx]

##### Additional file 2 - Summary of included articles

|  | **Title** | **Author/Year (Bibliography)** | **Type of Study** | **Methodology** | **Purpose of the study** | **Population characteristics** | **Key findings** | **Proposed solutions** |
| --- | --- | --- | --- | --- | --- | --- | --- | --- |
| 1 | A framework for mentoring of medical students: thematic analysis of mentoring programmes between 2000 and 2015 | Tan, Y. S., et al. (2018). "A framework for mentoring of medical students: thematic analysis of mentoring programmes between 2000 and 2015." Advances in Health Sciences Education 23(4): 671-697. | Systematic review | Five authors (JHS, PYY, LK, TYS, AT) to carried out independent searches of guidelines and accounts of novice mentoring published between 1 January 2000 and 31 December 2015 in the ERIC, Embase, Web of Science, PubMed, Cochrane Database of Systematic Reviews, CINAHL, OVID and Science Direct databases. The authors were guided by the Best Evidence Medical Education (BEME) guidelines for reference (Haig and Dozier 2003) and aided by two librarians. | To explore the characteristics of mentoring's nature, the distinctiveness of prevailing mentoring approaches, and the dominance of mentoring between a senior clinician and a medical student. This study hopes to ensure a consistent approach to novice mentoring in both undergraduate and postgraduate medical schools in Singapore. | Medical students and mentors in all clinical specialties within the medical school curricular | 1. Thematic analysis of the 6 cross sectional studies, 27 case studies and 1 commentary revealed 4 themes—preparation, initiating and supporting the mentoring process and the obstacles to effective mentoring. 2. There is a need to balance consistency in mentoring approaches whilst retaining flexibility to meet the individual needs of mentees and mentors across the mentoring journey. | The Mentoring Framework inculcates evidence-based recommendations to bridge gaps in practice and provide consistent guidance across the various phases of the men- toring process. This framework allows for differences in setting, healthcare systems and culture and serves to operationalise an effective program around the key elements of successful mentoring programs. |
| 2 | A systematic review of qualitative research on the meaning and characteristics of mentoring in academic medicine | Sambunjak, D., et al. (2010). "A systematic review of qualitative research on the meaning and characteristics of mentoring in academic medicine." Journal of general internal medicine 25(1): 72-78. | Systematic review | We included studies that used qualitative research methodology to explore the meaning and characteristics of mentoring in academic medicine. Two investigators independently assessed articles for relevance and study quality, and extracted data using standardized forms. No restrictions were placed on the language of articles. A total of 8,487 citations were identified, 114 full text articles were assessed, and 9 articles were selected for review. All studies were conducted in North America, and most focused on the initiation and cultivation phases of the mentoring relationship. | Mentorship is perceived to play a significant role in the career development and productivity of academic clinicians, but little is known about the characteristics of mentorship. This knowledge would be useful for those developing mentorship programs. To complete a systematic review of the qualitative literature to explore and summarize the development, perceptions and experiences of the men- toring relationship in academic medicine. | Medical students or physicians at a medical school, university hospital or academic general practice. | 1. Men- toring was described as a complex relationship based on mutual interests, both professional and personal. 2. Mentees should take an active role in the formation and development of mentoring relationships. 3. Good mentors should be sincere in their dealings with mentees, be able to listen actively and understand mentees' needs, and have a well-established position within the academic community. 4. Some of the mentoring functions aim at the mentees’ academic growth and others at personal growth. 5. Barriers to mentoring and dysfunctional mentoring can be related to personal factors, relational difficulties and structural/institutional barriers. | 1. Training and education. 2. Fostering relationship. 3. Choice and availability of mentors. 4. Mentoring reward. 5. Future research should focus more on the separation and redefinition phases of mentoring relationship and use a variety of qualitative meth- odologies and approaches to expand and deepen the body of knowledge about this important, yet elusive phenomenon. |
| 3 | Advancing institutional efforts to support research mentorship: a conceptual framework and self-assessment tool | Keyser, D. J., et al. (2008). "Advancing institutional efforts to support research mentorship: a conceptual framework and self-assessment tool." Acad Med 83(3): 217-225. | Discussive | We begin by describing how institutions can shape the key domains of research mentorship, then we describe a self-assessment tool for monitoring policies. | Few studies have offered details as to how  precisely institutions should conduct research mentoring. | Research institutions | Systematic empirical evidence on mentoring is limited and cannot be used “to suggest mentorship strategies that should be implemented at academic institutions.” | 1. The mentor should have knowledge, interest, skills and resources in the mentee’s specific area of research. 2. Institutions should incentivise mentorship. 3. Mentees should be guided on responsible research via direct communication and indirect observation of mentors. 4. Institutions should dedicate time during mentorship to discuss professional development of mentees and mentors in research careers. |
| 4 | Attracting Surgical Clerks to Surgical Careers: Role Models, Mentoring, and Engagement in the Operating Room | Berman, L., et al. (2008). "Attracting Surgical Clerks to Surgical Careers: Role Models, Mentoring, and Engagement in the Operating Room." Journal of the American College of Surgeons 207(6): 793-800.e792. | Cross-sectional study (qualitative study) | Third-year medical students completed an online survey after their surgery clerkship. The data were collected between September 2006 and September 2007. | With the decline of medical students who choose careers in surgery in recent years, this study seeked to assess medical students’ level of interest in surgery at the end of their clerkship and their impressions of surgeon role-modelling and mentorship | Surgery; Medical students | 1. Students were more likely to express interest in a career in surgery if they had hands-on experience in the operating room, strong mentorship, and positive role models, during the surgical clerkship. 2. There seems to be no substitute for operating room participation - laparoscopic simulation exercises, involvement in patient care outside of the operating room did not increase students' interest in a career in surgery. | To optimize students’ clerkship experiences and attract top candidates to the field of surgery, clerkship directors should encourage meaningful engagement of students in the operating room and facilitate mentoring experiences. |
| 5 | Being a mentor for undergraduate medical students enhances personal and professional development | Stenfors-Hayes, T., et al. (2010). "Being a mentor for undergraduate medical students enhances personal and professional development." Med Teach 32(2): 148-153. | Cross-sectional study (qualitative study) | Data was gathered through a questionnaire to all 83 mentors (response rate 75%) and semi-structured interviews with a representative sample of 10 mentors. | There is increasing evidence of the positive effects of mentoring in medical undergraduate programmes. This study aims to describe the effects of mentoring on mentors. | Mentors to undergraduate medical students | 1. Findings show, for example, that a majority of respondents developed their teaching as a result of their mentorship and improved their relations with students.  2. Most respondents claimed that being a mentor led to an increased interest in teaching and increased reflections regarding their own values and work practices. 3. Being a mentor was perceived as rewarding and may lead to both personal and professional development. | 1. Other studies are needed to further explore transferability of our findings (Graneheim & Lundman 2004), that is if similar relationships and benefits of being a mentor exist in other areas. |
| 6 | Characteristics of successful and failed mentoring relationships: a qualitative study across two academic health centers | Straus, S. E., et al. (2013). "Characteristics of successful and failed mentoring relationships: a qualitative study across two academic health centers." Academic medicine : journal of the Association of American Medical Colleges 88(1): 82-89. | Cross-sectional study (qualitative study) | The authors completed a qualitative study through the Departments of Medicine at the University of Toronto Faculty of Medicine and the University of California, San Francisco, School of Medicine between March 2010 and January 2011. They conducted individual, semistructured interviews with faculty members from different career streams and ranks and analyzed transcripts of the interviews, drawing on grounded theory. The authors completed interviews with 54 faculty members. | To explore the mentor–mentee relationship with a focus on determining the characteristics of effective mentors and mentees and understanding the factors influencing successful and failed mentoring relationships. | Medical faculty | 1. Characteristics of effective mentors and mentees. 2. Actions of effective mentors. 3. Characteristics of successful and failed mentoring relationships. 4. Tactics for successful mentoring relationships. 5. Successful mentoring relationships were characterized by reciprocity, mutual respect, clear expectations, personal connection, and shared values. Failed mentoring relationships were characterized by poor communication, lack of commitment, personality differences, perceived (or real) competition, conflicts of interest, and the mentor’s lack of experience. | 1. Future studies must address the association between a failed mentoring relationship and a faculty member’s career success, how to assess different approaches to mediating failed mentoring relationships, and how to evaluate strategies for effective mentorship throughout a faculty member’s career. |
| 7 | Cultural implications of mentoring in sub-Saharan Africa: A qualitative study | Sawatsky, A., et al. (2016). "Cultural implications of mentoring in sub-Saharan Africa: A qualitative study." Medical Education 50: 657-669. | Cross-sectional study (qualitative study) | A qualitative study using in-depth, semi-structured interviews and thematic analysis was conducted to explore the meaning of mentorship at the study institution. | To explore the role of culture in the development and maintenance of mentoring relationships within the context of the University of Malawi College of Medicine. | Medical students, interns, registrars and faculty members | 1. Themes were identified within three domains: the intrapersonal; the interpersonal, and the institutional.  2. Intrapersonal themes included Malawian politeness, mentoring needs, and friendliness and willingness to help.  3. Interper- sonal themes included understanding the role of the mentor, respect for elders, personal and professional boundaries, and perceptions of others.  4. Institutional themes included the super- visor versus mentor, time pressures, tension about the scope of training, and the mentoring cycle. | 1. The authors propose an updated model for mentoring in academic medicine. This model can inform future research on mentoring and may serve as a model in the larger effort to provide faculty development in mentoring across sub-Saharan Africa. |
| 8 | Enhancing medical students' reflectivity in mentoring groups for professional development - a qualitative analysis | Lutz, G., et al. (2017). "Enhancing medical students' reflectivity in mentoring groups for professional development - a qualitative analysis." BMC medical education 17(1): 122-122. | Cross-sectional study (qualitative study) | A qualitative design was applied using semi-structured focus group interviews with preclinical students and semi-structured individual interviews with mentors and co-mentors. The interview data were analyzed using thematic content analysis. | This study explores both the attitudes of those students towards a group mentoring program involving all preclinical students as well as faculty members and co-mentoring clinical students and factors that might hinder or enhance how students engage in reflective discourse. | Preclinical medical students, faculty members and co-mentoring clinical students | 1. A well-designed and empathetic setup of group mentoring programs can help raise openness towards engaging in meaningful reflective discourse.  2. Reflection on and communication of professional challenges can, in turn, improve professional development, which is essential for high quality patient care. | 1. The topic of positive engagement in reflection on the professional intra-and interpersonal or institutional challenges faced during students' studies could be studied from three perspectives, the mentors’, the co-mentors’ and the mentees’. This would allow a detailed understanding of the objections to such experi- ences and the setup factors that might be helpful in letting students engage in the group process. |
| 9 | Ethical Implications In Mentoring Medical Students | Usmani A, S. S., Omaeer Q. (2011). "Ethical Implications In Mentoring Medical Students." Asian Human Rights Commission. | Cross-sectional study (qualitative study) | A questionnaire based survey was conducted. All the mentors were recruited which were 22 in number and there was 100% response. The questionnaire which was distributed among the faculty members consisted of close-ended and open-ended questions. The answers which needed elaboration were asked in open-ended questions. | The mentor-mentee program in Bahria University Medical and Dental College has many benefits, but along with its success there are some ethical issues which need to be recognized and identified. This paper seeks to identify ethical implications of mentoring medical students. | Medical students in Bahria University Medical and Dental College | 1. The structured mentoring program has been proved to be very successful, however it has been seen that at certain times there is violation of the close interpersonal relationship among mentor and mentee.  2. Some mentors have shown lack of interest in mentoring due to which they do not give proper time to their mentee. Breach in confidentiality and privacy of the mentee has also been noticed.  3. A very common issue is the paternalistic approach of the mentors towards their mentee and this attitude may pose to be harmful. | 1. Respecting privacy and to safeguard ones confidentiality are hence of utmost importance in order to maintain integrity of the process of mentoring and this must under all circumstances be observed all the time.  2. It is an obligation of all mentors to avoid engaging in multiple relationships because this may result in impairment of their ability to perform their jobs properly and ethically, also it may cause harm or exploit the mentee.  3. A mentor is one who has strong belief in autonomy and helps in developing his or her mentees’ personality. |
| 10 | Expanding the Orthopaedic Pipeline: The B.O.N.E.S. Initiative | Earp, B. E. and T. D. Rozental (2019). "Expanding the Orthopaedic Pipeline: The B.O.N.E.S. Initiative." Journal of Surgical Education. | Observational study | A workshop was designed to introduce female medical students in the New England region to the technical aspects of orthopaedics as well as bring them into contact with a dynamic group of female mentors. | Orthopaedic surgery continues to struggle in attracting women and under-represented minorities to the field despite increasing numbers of female medical school graduates. The workshop hopes to tackle the barriers of limited exposure during medical school and the scarcity of female mentors. | Female medical students interested in orthopaedics | 1. 97% of 155 female medical students par- ticipated in the program over 3 years found it useful. 2. 22 of 59 students who have since become eligible for the match, matched into an orthopaedic surgery resi- dency.  3. 22% of 36 match eligible first and second year participants matched in orthopaedic surgery while 61% of 23 match eligible third and fourth year student attendees successfully matched in orthopaedic surgery programs. | Encouragement of programs to consider similar outreach initiatives to enhance students' interest in the field. |
| 11 | Female Medical Student Retention in Neurosurgery: A Multifaceted Approach | Dixon, A., et al. (2019). "Female Medical Student Retention in Neurosurgery: A Multifaceted Approach." World Neurosurgery 122: 245-251. | Cross-sectional study (qualitative study) | A comprehensive survey was created and distributed to the 2017-2018 Rutgers New Jersey Medical School student body, requesting anonymous input from female medical students. | To further explore factors that deter female medical students from pursuing neurosurgery. | Female medical students | 1. 26.9% of 104 respondents had considered neurosurgery as a career and felt dissuaded because of their gender. 2. 88% did not have a senior female medical student pursuing neurosurgery or a female neurosurgical resident as a mentor. More than half of respondents disagreed that they would be dissuaded from a field if they did not have a female mentor. 3. The 88.46% of women who felt that there was a glass ceiling in medicine were also more likely to feel that they would face inequality and adversity that would inhibit training in a male-dominated field. | 1. Societal and cultural boundaries, such as family planning and gender roles, must be contested so that women are reassured that their careers will not be negatively impacted in the future. 2. Residency malignancy should be pre- vented, while increasing female mentorship by both male and female neurosurgeons. 3. The neurosurgical community should expand on our survey results in a productive manner for further implementation of interventions that can retain women in this field at the level of the medical student. |
| 12 | Fostering research skills in undergraduate medical students through mentored students projects: example from an Indian medical school | Devi, V., et al. (2010). "Fostering research skills in undergraduate medical students through mentored students projects: example from an Indian medical school." Kathmandu Univ Med J (KUMJ) 8(31): 294-298. | Systematic review | Mentored Student Project was implemented in the curriculum during second year of MBBS programme with the intention of developing research skills essential to the career development of medical students. A questionnaire (Likert’s five point scale) on students’ perceptions regarding improvement in research skills was administered to medical students after they had completed their Mentored Student Project . | This study examines students’ perceptions regarding research skills improvement after participating in the Mentored Student Project programme at Melaka Manipal Medical College, Manipal Campus, India. Additionally, this paper describes the initiatives taken for the continual improvement of the Mentored Student Project programme based on faculty and student perspectives. | Second year medical students from Malaka Manipal Medical College | 1. The present study revealed that the MSP programme not only increased students’ research skills as evident from their perceptions but was also successful in fostering a positive attitude towards scientific research. 2. The present study also provided scope for further refinement of the MSP programme based on students’ and faculty perspectives. | 1. The authors are planning to research on more objective measures of the MSP programme. |
| 13 | How Assigned Faculty Mentors View their Mentoring Relationships: An Interview Study of Mentors in Medical Education | Dobie, S., et al. (2010). "How Assigned Faculty Mentors View their Mentoring Relationships: An Interview Study of Mentors in Medical Education." Mentoring & Tutoring: Partnership in Learning 18(4): 337-359. | Cross-sectional study (qualitative study) | As part of an evaluation process for the newly formed colleges, faculty mentors were inter- viewed early in the second and third years of the colleges program. The researchers analyzed the data from the second round of individual interviews with 29 college mentors when specific questions about mentoring were asked. Interviews with college mentors were semi-structured, lasted 30 minutes on average, and elicited responses to how the mentors worked with students and their perceptions of the benefits and challenges of this work. | This qualitative study of 29 physician mentors reports their perspectives on mentoring medical students in a well-respected medical school’s formal, assigned, longitudinal mentoring program that has a curricular component in the second year. | Faculty physician mentors | 1. An assigned mentoring program for medical students can have benefit and be satisfying to the mentors. 2. College mentors were responsible for tracking student progress through medical school. This accountability may have served to provide students with the focused support found to be lacking in prior reports.  3. Having funded time to mentor and teach students, as well as being part of a learning community, were important factors in mentor satisfaction in this program. | 1. More research is needed on the impact on mentees or mentors from established medical school mentoring programs.  2. Further work to examine the longitudinal professional development of the college mentors would also be informative. |
| 14 | Informal mentoring between faculty and medical students | Rose, G. L., et al. (2005). "Informal mentoring between faculty and medical students." Acad Med 80(4): 344-348. | Perspective paper | Not explicitly stated though it would appear that this is an editorial built upon a literature review. | The authors describe the development of optimal mentoring rela- tionships, emphasizing the importance of experience and flexibility in working with beginning to advanced students of dif- ferent learning styles, genders, and races. | Medical students and faculty mentors | 1. Good mentoring relationships involve self-knowledge and identification of one’s style and limitations. 2. Mentors should be clear with trainees about what they can and cannot offer; part of the role is knowing when to refer someone to another resource that might be more helpful. 3. Mentoring also entails familiarity with the protege's sociocultural background and stage of educational development, discussion of goals, and awareness of how the structure of the relationship impacts its dynamics. 4. Good mentoring relationships require active maintenance.  5. A good mentor maintains sight of the overall goal of the relationship and holds the focus for the mentee’s personal and professional growth over time. | None |
| 15 | Last Laughs: Gallows Humor and Medical Education | Piemonte, N. M. (2015). "Last Laughs: Gallows Humor and Medical Education." J Med Humanit 36(4): 375-390. | Opinion | Not explicitly stated though it would appear that this is an editorial built upon a literature review. | This paper argues that "backstage" gallows humor among clinical mentors is symptomatic and indicative of the failure in medicine to attend fully to the complexity and profundity of the lived experiences of illness, suffering and death,. | Clinical mentors | 1. It is the author's view that reorienting the conversation toward the reasons why students and doctors feel the need to use such humor in the first place offers richer insight into this issue. 2. When mentors fail to help students tread the high-stakes, life-and-death, morally sensitive world of medicine by engaging in gallows humor, applying the framework of moral injury to medical education does not seem like too far a stretch. 3. Gallows humor and other behaviours inconsistent with the espoused ideals of professionalism are embedded and perpetuated within the tacit culture of medicine - it has significant indluence on student's understanding of professionalism in medicine compared to official curriculum.  4. Gallows humor may be used as a way to cope with topics too heavy to face and reflect upon directly | 1. There is a desperate need for a change in medical culture and education which requires a rethinking of medical education and consideration of the personal qualities it cultivates. 2. We must, infuse medical education with other ways of knowing, thinking, and relating, and a space should be created for physicians and physicians-in-training to learn about and reflect upon the distressing and painful issues that can arise when caring for sick patients and their families. |
| 16 | Longitudinal mentorship to support the development of medical students' future professional role: a qualitative study | Kalen, S., et al. (2015). "Longitudinal mentorship to support the development of medical students' future professional role: a qualitative study." BMC medical education 15: 97. | Cross-sectional study (qualitative study) | Sixteen medical students at a Swedish university were interviewed individually about their experiences of combined group and one-to-one mentoring that is given throughout their studies. The mentoring programme was focused on the non-medical skills of the profession and used CanMEDS roles of a physician for students’ self-assessment. Data were analysed using a latent, interpretive approach to content analysis. | Mentoring has been employed in medical education in recent years, but there is extensive variation in the published literature concerning the goals of mentoring and the role of the mentor. The aim of this qualitative study is to explore how formal and longitudinal mentoring can contribute to medical students’ professional development. | Medical students in Sweden | 1. The mentorship enabled the students to create a view of their future professional role and to integrate it with their own personalities.  2. The students’ understanding of professional competence and behaviour evolved during the mentorship and they made advances towards understanding the wholeness of the profession.  3. This approach to mentorship supported different components of the students’ professional development. | 1. Formalized and longitudinal mentoring focusing on the non-medical skills can be recommended to help medical students to integrate their professional role with themselves as individuals and promote understanding of professional competence in the process of becoming a physician. |
| 17 | Making the most of mentors: a guide for mentees | Zerzan, J. T., et al. (2009). "Making the most of mentors: a guide for mentees." Acad Med 84(1): 140-144. | Perspective paper | Not explicitly stated though it would appear that this is an editorial built upon a literature review. | Many papers focus on mentoring from the mentor’s perspective, but few give guidance to mentees forging these critically important relationships. The authors review the initiation and cultivation of a mentoring relationship from the perspective of a mentee at any stage, and they propose specific strategies for mentee success. | Medical students to junior faculty | 1. When a mentee knows him- or herself, knows his or her values and needs, manages up, makes the relationship a high priority, and shows appreciation, he or she will most likely become successful. 2. | 1. We outline the strategy, managing up, which may be helpful to improve mentoring relationships. 2. By implementing these specific tasks, mentees can nurture and improve a relationship that may ultimately become a productive and enjoyable force in the careers of both the mentor and the mentee. |
| 18 | Medical Student Mentorship in Plastic Surgery: The Mentee’s Perspective | Barker, J. C., et al. (2016). "Medical Student Mentorship in Plastic Surgery: The Mentee’s Perspective." Plastic and Reconstructive Surgery 137(6): 1934-1942. | Cross-sectional study (qualitative study) | A 30-question electronic survey was sent to all recently matched integrated track postgraduate year–1 residents regarding their experience with mentorship as a medical student. The survey consisted of five general areas of questions - (1) mentee and mentor demographic information, (2) how mentor-mentee relationships are formed, (3) how mentor-mentee relationships are maintained, (4) qualities sought in a mentor and the benefits to mentees, and (5) barriers to mentorship from the mentee’s perspective. | As dedicated research devoted specifically to the value and impact of mentorship toward medical students is lacking, this article seeks to evaluate medical student mentorship in plastic surgery from the medical student perspective. | Plastic surgery; Medical students | 1. Interactions between the mentee and the mentor optimally occur in a one-on-one environment. 2. The content of the mentoring experience is different from standard educational content - students reported relying on mentors for career advice and learning to navigate the waters of the specialty. 3. Effective mentorship requires time and commitment, not prerequisites or specific skill sets. | 1. Encouragement and facilitation of resident mentorship of medical students could provide an opportunity for both acquisition of skills by residents and also improved mentoring of medical students in plastic surgery. 2. The pursuit for increased awareness, innovative solutions, and quality improvement in mentorship must continue. Successful mentorship of medical students may contribute to the future of plastic surgery, and a commitment toward this endeavor is needed at the local, departmental, and national leadership levels. |
| 19 | Medical Student Mentorship in Plastic Surgery: The Mentor’s Perspective | Janis, J. E. and J. C. Barker (2016). "Medical Student Mentorship in Plastic Surgery: The Mentor’s Perspective." Plastic and Reconstructive Surgery 138(5): 925e-935e. | Cross-sectional study (qualitative study) | A 25-question electronic survey was sent to all active members of ACAPS and AAPS regarding their experience with mentorship of medical students. The survey consisted of five general areas of questions that included the following: (1) mentee and mentor demographic information, (2) how mentormentee relationships are formed, (3) how mentormentee relationships are maintained, (4) qualities sought in a mentee and the benefits to the mentor, and (5) barriers to mentorship from the mentor’s perspective. | The importance and value of mentorship is commonly discussed in the plastic surgery literature, however, the views on mentorship specifically from the mentor’s perspective, has not been as thoroughly evaluated and reported. | Plastic surgery | 1. Due to increasing emphasis placed on work productivity, administrative and academic time obligations, and difficult work-life balance issues, attending surgeon mentors preferred less frequent and less personal interactions, as compared to what mentees would have preferred. | 1. With ever-increasing demands that place plastic surgery faculty at risk for burnout and lack of faculty retention, perhaps it is equally as important to emphasize, promote, and support activities, such as mentoring and teaching, that are proven to reduce these risk factors. 2. The following suggestions were proposed for medical student mentorship in plastic surgery: (i) Provide early exposure; (ii) Provide formal opportunities for career exploration; (iii) Tailor the content of interactions toward personal and career development; (iv) Initiative and prioritizing mentoring on an individual level yield the greatest mentoring relationships. |
| 20 | Medical students’ perceptions of mentoring: a focus-group analysis | Hauer, K. E., et al. (2005). "Medical students’ perceptions of mentoring: a focus-group analysis." Medical Teacher 27(8): 732-734. | Observational study | The authors conducted four focus groups lasting 60–90 minutes, each with four to eight fourth-year students with or without mentors who responded to an invitation to participate and were able to attend. Participants completed a demographic questionnaire. Data were analyzed using grounded theory. | Characteristics of medical students’ mentoring relationships and factors that affect mentoring during medical school are poorly understood. The authors conducted four focus groups with fourth-year medical students to explore what students sought from mentors, perceived barriers to mentoring and suggestions for improvement. | Fourth year medical students from University of California | 1. The short duration of courses, abrupt change from classroom learning to clerkships and limited exposure to clinicians were perceived as barriers to mentoring. 2. Students recommended that the school explicitly promote mentoring with early education about finding mentors, placing the responsibility on students while also expanding the pool of potential mentors. 3. It is concluded that medical students characterize mentoring in terms of the inter- personal dynamics of the relationship, emphasizing personal connection and advocacy. | 1. Educating and empowering students along with faculty education regarding students’ needs may improve mentoring. |
| 21 | Mentoring for first year medical students: humanising medical education | Bhatia, A., et al. (2013). "Mentoring for first year medical students: humanising medical education." Indian J Med Ethics 10(2): 100-103. | Observational study | After needs analysis of students and faculty, a small-group mentoring programme for new medical students was initiated. At year-end, feedback was collected using an open-ended questionnaire. | New entrants are vulnerable to the challenges of the medical course; mentoring programmes are known to offer support. This paper evaluated the experiences of students and faculty enrolled in a new mentoring programme. | First year medical students | 1. There was no contact in one-third of the cases; the commonest reasons cited were lack of mentee initiative, time and commitment.  2. Over 95% of respondents believed that mentoring was a good idea; many believed the mentee benefitted; mentors also reported improved communication and affective skills; 60 (77.0%)  3. Mentoring may be a means of honing the affective domain and humanitarian instincts of medical faculty and students. | 1. Depending on needs and cultural sensitivities, each institution should be encouraged to evolve its own mentoring programme. |
| 22 | Mentoring in the Era of #MeToo | Byerley, J. S. (2018). "Mentoring in the Era of #MeToo." JAMA 319(12): 1199-1200. | Perspective paper | Not applicable. | In the wake of the inspiring but incredibly upsetting #MeToo movement, I find myself concerned not only for the women who have already experienced sexual harassment, and certainly there are many, but also for those yet to come. Some will unfortunately experience the same pain, fear, humiliation, and stigma of sexual harassment as the stories being brought to light by the brave women who have been reporting their experi- ences today. In addition, I worry about the potential for gender-based neglect, an unintended backlash in response to this bright light. | Not applicable. | 1. Behaviours mentors demonstrate to make mentees feel safe: exemplary professional behavior, demonstrating integrity, having warm personalities and refraining from physical touch, avoid generalizing comments about gender. 2. Empowered male mentors see this big picture, and they are also prepared to respond when they observe sexist behavior. When male leaders respond actively to sexist situations progress is made. When this type of concrete support is consistently demonstrated to mentees, the trust of the mentoring relationship flourishes and the mentee moves toward her full potential. | 1. Men with power must name the issue of sexual harassment and make it clear that harassing behavior is never acceptable, and also invite the mentee to call out behavior that causes discomfort in any way. Men who openly address the issue of mistreatment of women in a confident and respectful manner empower women and advance our organizations to be more inclusive and productive work environments. 2. Men should spark such conversations about treatment of women in groups with power and authority. The message of leaders must be that sexual harassment will not be tolerated and diversity is a core value. 3. Professional behavior is key to establishing a safe environment for effective mentoring. 4. A diverse community of leaders can create a more supportive, respectful culture. To that end, building mentoring relationships across lines that sometimes divide us must be encouraged. |
| 23 | Mentoring medical students during clinical courses: A way to enhance professional development | Kalén, S., et al. (2010). "Mentoring medical students during clinical courses: A way to enhance professional development." Medical Teacher 32(8): e315-e321. | Observational study | Medical students (n 1⁄4 118) during their third and fourth years of their studies were offered a personal mentor for 2 years and followed up via a questionnaire when the mentoring programme was completed. Statistical software was used to compute data. Open-ended questions were analyzed by content analysis. | Only a few structured mentoring programmes for medical students have been reported in the literature. The objective of this study was to investigate undergraduate medical students’ experiences and perceptions of one-to-one mentoring and whether they felt that the mentorship promoted their personal and professional development. | Third and fourth year medical students | 1. Most of the respondents experienced that the mentoring programme had facilitated their professional and personal development. 2. The role of the mentor was experienced as being more supportive than supplying knowledge. 3. The students appreciated talking to a faculty not connected with their courses.  4. The few barriers to a successful mentorship were mainly related to timing logistics and ‘personal chemistry’. | 1. One-to-one mentoring during clinical courses seems to enhance the medical student’s professional and personal development.  2. Future studies are needed to get a deeper understanding and knowledge about factors of importance for successful mentorship. |
| 24 | Mentoring medical students in academic emergency medicine | Garmel, G. M. (2004). "Mentoring medical students in academic emergency medicine." Acad Emerg Med 11(12): 1351-1357. | Perspective paper | Not explicitly stated though it would appear that this is an editorial built upon a literature review. | This article will describe the role of the mentor, suggest ways to increase the likelihood of successful mentoring, and identify pitfalls in the mentoring process predominantly related to medical students. | Medical students in academic emergency medicine | 1. A healthy mentoring relationship is likely to strengthen one’s moral compass, which is essential to continued learning and self- improvement. Ideally, all students and physicians, no matter how senior, should be in such a relationship and have the opportunity to benefit from this component of medical education and growth. 2. The investment of time through mentoring medical students is likely to influence the specialty of EM, because some of these students will develop into future leaders. | 1. Providing students with productive and meaningful mentoring relation- ships sets an example for future mentors and may be translated into successful generations of physician mentors. |
| 25 | Mentoring programs for medical students - A review of the PubMed literature 2000-2008 | Frei, E., et al. (2010). "Mentoring programs for medical students - A review of the PubMed literature 2000-2008." BMC medical education 10: 32. | Systematic review | A PubMed literature search was conducted for 2000 - 2008 using the following keywords or their combinations: mentoring, mentoring program, medical student, mentor, mentee, protégé, mentorship. Although a total of 438 publications were identified, only 25 papers met the selection criteria for structured programs and student mentoring surveys. | Within the framework of planning a mentoring program for medical students at Zurich University, an investigation was carried out into what types of programs exist, what the objectives pursued by such programs are, and what effects are reported. | Medical students | 1. The mentoring programs reported in 14 papers aim to provide career counseling, develop professionalism, increase students' interest in research, and support them in their personal growth.  2. There are both one-to-one and group mentorships, established in the first two years of medical school and continuing through graduation. The personal student-faculty relationship is important in that it helps students to feel that they are benefiting from individual advice and encourages them to give more thought to their career choices.  3. A mentor should empower and encourage the mentee, be a role model, build a professional network, and assist in the mentee's personal development. A mentee should set agendas, follow through, accept criticism, and be able to assess performance and the benefits derived from the mentoring relationship. | 1. Europe, more mentoring programs should be developed, but would need to be rigorously assessed based on evidence of their value in terms of both their impact on the career paths of juniors and their benefit for the mentors. Medical schools could then be monitored with respect to the provision of mentorships as a quality characteristic. |
| 26 | Mentoring relationships between senior physicians and junior doctors and/or medical students: A thematic review | Sng, J. H., et al. (2017). "Mentoring relationships between senior physicians and junior doctors and/or medical students: A thematic review." Med Teach 39(8): 866-875. | Systematic review | Literature search was performed on publications across PubMed, ERIC, Cochrane Database of Systematic Reviews, OVID and ScienceDirect databases between 1 January 2000 and 31 December 2015 by three independent reviewers. The BEME guide and STORIES statement were used to develop a narrative from the articles selected. | Mentoring relationships are pivotal to the outcome of the mentoring process. This thematic review seeks to study the key aspects of mentoring relationships between senior physicians and junior doctors and/or medical students to inform efforts to improve mentoring programs. | Medical students, junior doctors | 1. Embrace of a consistent mentoring approach to ensure effective oversight of the mentoring process must be balanced with sufficient flexibility to ensure a mentee-centered approach. 2. Efforts must be made to optimize the key aspects of mentoring relationships in order to ensure successful mentoring processes and outcomes. | 1. It is hoped that better evidenced-based understanding of the key elements of mentoring relationships will inspire such balanced mentoring programs and encourage more holistic, systematic and longitudinal research on mentoring that will better inform the creation of mentoring theories, mentoring guidelines and ethical frameworks for mentoring programs in medicine. |
| 27 | Mentoring undergraduate medical students: experience from Bahria University Karachi | Usmani, A., et al. (2011). "Mentoring undergraduate medical students: experience from Bahria University Karachi." J Pak Med Assoc 61(8): 790-794. | Cross-sectional study (qualitative study) | An anonymous data collection tool was distributed to 22 faculty members trained as mentors at Bahria University Medical and Dental College, Karachi, Pakistan. | To explore the perceptions and the effects on mentors regarding mentoring medical students at Bahria University Medical and Dental College, Karachi, Pakistan. | Medical students at Bahria University Medical and Dental College, Karachi, Pakistan. | 1. Majority of the mentors rate themselves as "good" or "satisfactory" mentors (55% and 45%) 2. The mentors give enough time to their mentees and are comfortable with any gender or culture, and help their mentees settle down and improve them academically as well as personally (86.36%). 3. Half the mentors sacrifice their personal time for the development of their mentees. 4. 59% of mentors felt that their mentees showed good improvement academically due to these sessions, while 41% said that they were satisfied with the academic results of their mentees. 5. The perception of mentors about the structured mentoring programme subjectively appears to be a promising strategy for young medical students. Objective assessment of these mentors is needed. | None |
| 28 | Mentors' perspectives on group mentorship: a descriptive study of two programs in child and adolescent psychiatry | Alleyne, S. D., et al. (2009). "Mentors' perspectives on group mentorship: a descriptive study of two programs in child and adolescent psychiatry." Acad Psychiatry 33(5): 377-382. | Observational study | Similar group mentorship programs were imple- mented at two child and adolescent psychiatry conferences, one national and the other international. The program included three daily small group meetings, one closing meeting for all partici- pants, and administration of a web-based survey. | The authors assess mentors’ perceptions of mentor- ing and experiences participating in an intensive, small-group mentorship program, with particular attention to potential chal- lenges in their retention and the recruitment of new mentors to similar, future programs. | Medical students, psychiatric residents, child and adoles- cent psychiatry fellows, and junior faculty | 1. Mentors found the intensive group co-mentorship model to be a powerful, time-efficient, and enjoyable ap- proach, although group composition, schedule coordina- tion, and logistics remain challenges for future programs. | 1. The mentors’ positive perceptions bode well for mentor recruitment and retention in similar mentorship programs and suggest that the program might also be attempted in other medical fields.  2. Given concerns about the shortage of mentors and the lack of time to mentor, this model may be used as a complement or an alternative to the traditional individual mentorship model seen at academic institutions. |
| 29 | Mentorship in Medicine and Other Health Professions | Henry-Noel, N., et al. (2019). "Mentorship in Medicine and Other Health Professions." Journal of Cancer Education 34(4): 629-637. | Systematic review | The search used for the present literature review was conduct- ed in Ovid MEDLINE® database covering the period from 1946 to January Week 2 2018 database. Both subject headings and key words were utilized for the terms. The outcomes were further limited to articles published since 2013 and solely in English. | To evaluate the current literature on mentorship in academic medicine with focus on dif- ferent approaches to mentorship, mentor and mentee roles and responsibilities, mentor and mentee benefits, interprofessional collaboration, and mentorship in respect to gender and culture | Mentors and mentees in academic medicine | 1. Mentorship is an essential process in academic medicine. 2. The benefits of mentoring are not limited to the mentee but also extend to the mentor with respect to professional satisfaction and institutional recognition.  3. Mentoring helps maintain positive associations with residents and faculty members. | 1. Women in academia as well as ethnic minorities entering into medicine and allied health professions face systematic barriers to mentorship that should be acknowledged, and thus, access to mentors for women faculty members and from ethnic minority groups should be improved. 2. Future research must be conducted to investigate how these particular groups can be more successful allowing these positive shifts in the medical and other healthcare fields to continue. |
| 30 | Odysseus's lament: Death of mentor | Liang, T. J. (2000). "Odysseus's lament: Death of mentor." Gastroenterology 119(6): 1429. | Opinion | Not applicable. | The future of academic medicine is mired in uncertainty due to the recent sea changes in academic health centres and medical schools. Among the most worrisiome is the possibility that fewer of the best and brightest people will ener an academic track in future. | Medical students, junior doctors | 1. Many would agree that the people who had the most impact on our professional lives are those we considered as our mentors. As our scientific parents, role models, and ardent supporters, mentors are often responsible for our success in academic life. 2. Regrettably, the fundamental tenets of mentorship have been eroded by the current formula for tenure and pro- motion in academic medicine. Traditional class room and one-on-one teaching has sometimes been traded for the impersonal presence of a monitor and keyboard, further straining this al- ready fragile relationship. 3. The author believes that the path of success rests on a solid foundation of strong mentorship start- ing early in one’s career development. | 1. Faculty mem- bers must be recognized and awarded by their medical schools and academic institutions for their commitment to mentoring, which is often not consid- ered in the equation for promotion and tenure. 2. As we chart the course of our profes- sion in uncertain water, we must strive to preserve the mentor-apprentice para- digm. |
| 31 | Operating Room Assist: Surgical Mentorship and Operating Room Experience for Preclerkship Medical Students | Cloyd, J., et al. (2008). "Operating Room Assist: Surgical Mentorship and Operating Room Experience for Preclerkship Medical Students." Journal of Surgical Education 65(4): 275-282. | Observational study | The elective paired 36 first-year students with 24 surgeons and instructed students to participate in at least 2 surgeries over the 3-month elective period. Students, surgeons, and operating room nurses filled out questionnaires after each surgery. | Despite the importance of preclerkship experiences, surgical education has remained confined to the third-year operating room experience. Hence, a new clinical elective was created, in which preclerkship medical students were assigned a surgical mentor and invited into the operating room to assist in surgeries. | Surgery; Pre-clerkship Medical students | Of the 36 students, 6 students failed to enter the operating room, however, 30 students scrubbed and gowned for a total of 62 procedures during the elective period. Students consistently underrated their performance and contribution to the surgical team compared with the surgeons’ and nurses’ ratings. 75% of students also reported using a previously learned surgical skill during each surgery, proving the elective's success in allowing preclerkship medical students the opportunity to participate actively in the operating room. | 1. To tackle the issue of surgeon recruitment, potential surgical mentors should be reminded that (i) very little time commitment is involved; (ii) all students have been trained in basic surgical skills and operating room etiquette; (iii) surgeons will have the opportunity to teach students about surgery without having to evaluate the students. 2. An online evaluation system may be used in the future to increase participation. |
| 32 | Preventing neurophobia": Remodeling neurology education for 21st-century medical students through effective pedagogical strategies for "neurophilia"" | Shelley, B. P., et al. (2018). "Preventing "neurophobia": Remodeling neurology education for 21st-century medical students through effective pedagogical strategies for "neurophilia"." Annals of Indian Academy of Neurology 21(1): 9-18. | Perspective paper | Not explicitly stated though it would appear that this is an editorial built upon a literature review. | This article explores plausible factors that contribute to the genesis of neurophobia and multifaceted strategies to nurture interest in neurosciences and provide possible solutions to demystify neurology education, especially the need for evidence-based educational interventions. | Medical students in India | 1. The main impetus as far as the remedial measures are concerned would be to focus on the need for novel and flexible pedagogical teaching for reinventing effective and quality neurology education in the undergraduate medical curriculum. | 1. The fragmentation in the learning of basic neurosciences with clinical neurosciences should be tackled by integrating learning of basic neurosciences with early, effective, and multiple clinical exposures most efficiently under a neuro-mentorship program.  2. The need for robust educational research in order to generate the evidence to inform decision makers to initiate medical education reforms away from non-integrated, divisive neurology disccourse. |
| 33 | Redesigning a clinical mentoring program for improved outcomes in the clinical training of clerks | Lin, C. D., et al. (2015). "Redesigning a clinical mentoring program for improved outcomes in the clinical training of clerks." Med Educ Online 20: 28327. | Observational study | A redesigned clinical mentoring program was launched in a medical center according to previous theoretical and practical studies on clinical training workplaces. A four-wave web survey was conducted, comprising one evaluation of the former mentoring program and three evaluations of the redesigned clinical mentoring program. A structured and validated questionnaire encompassing 15 items on mentor performance and the personal characteristics of the clerks was used. Mixed linear models were developed for repeated measurements and to adjust for personal characteristics. | A lack of infrastructure in a mentoring program might deter relationship building between mentors and mentees. This study assessed the effect of a redesigned clinical mentoring program from the perspective of clerks, hoping to expound on the benefits of the redesigned program and identify potential improvements. | Medical students in clerkships | 1. Re- garding professional development, the mentees reported a steady improvement in mentor guidance in personal career interests, promotional opportunities, and coordinating professional goals. 2. The evaluation items regarding the mentors’ special, on-the-job coaching, the mentors’ ability to motivate others, the mentors’ devotion of additional time and consideration of the mentees’ careers, the mentees’ confidence in their mentors, and the mentees’ respect of their mentors’ knowledge of the accounting profession increased only 6 months after implementation. 3. Regarding personal support, the mentees rated sharing personal problems and dining after work progressively higher over time.  4. The mentees’ evaluation of how the mentors placed them on critical assignments and the other involved the mentors’ ability to teach others did not improve. | 1. The authors recommend the adoption of mentorship schemes for other cohorts of medical students and for different learning and training stages involved in becoming a physician. |
| 34 | Research Mentoring of Medical Students: A Win-Win | Kadom, N., et al. (2018). "Research Mentoring of Medical Students: A Win-Win." Journal of the American College of Radiology 15(12): 1771-1774. | Opinion | Not explicitly stated though it would appear that this is an editorial built upon a literature review. | There is often less effort spent on training medical students, perhaps because we think of it as a “waste of time” if these students later decide to go into a different specialty. This article seeks to elaborate on how there are shared goals and benefits when radiologists and medical students collaborate on research projects, and with attention to certain strategies and with a sup- portive infrastructure, this can be a mutually satisfying experience. | Medical students interested in radiology | 1. Mentoring can be limited to a project or last a lifetime, and it can build both academic and personal growth for both participants. 2. In-person meetings will be most beneficial for managing the project and building trust as the foundation of the mentoring rela- tionship. Although sometimes life gets in the way, both parties must commit to invest the time for the relationship to be successful. 3. Working with medical students on research projects has been one of the most rewarding experiences in my (N.K.) career as an academic radiologist. Being able to connect with young professionals, share the specialty of radiology with them, and build lasting mentor-mentee relationships, has given more mean- ing to my (N.K.) professional life. | None |
| 35 | Residents as Medical Student Mentors During an Obstetrics and Gynecology Clerkship | Sobbing, J., et al. (2015). "Residents as Medical Student Mentors During an Obstetrics and Gynecology Clerkship." Journal of graduate medical education 7(3): 412-416. | Observational study | A senior resident physician was assigned 1 to 2 medical students for a 6-week rotation. Students were provided MSMP information during clerkship orientation; residents were given information on MSMP requirements and were randomly assigned to students. We surveyed students and residents about their experience with the MSMP. | Resident physicians provide much of the clinical teaching for medical students during their clerkship rotations, but often receive no formal preparation or structure for teaching and mentoring students. The paper sought to evaluate a medical student mentoring program (MSMP) for students during their obstetrics and gynecology clerkship at a midwestern teaching hospital during the 2013–2014 academic year. | Medical students in Obstetrics and Gynecology clerkship | 1. Postclerkship, students indicated that they would participate in the MSMP again (32 of 38, 84%), and felt that having a mentor on other clerkships (30 of 36, 83%) would be beneficial. 2. Students reported receiving educational (20 of 41, 49%) and procedural (33 of 41, 80%) instruction, personal development feedback (23 of 41, 56%), and career advice (14 of 41, 34%) from resident mentors. 3. Residents did not feel burdened by students (14 of 17, 82%), and all responded that they would participate in the MSMP again. | 1. Feedback from medical students suggests that a mentoring program during clerkships may provide potential benefits for their careers and in 1-on-1 instruction. |
| 36 | Role models and mentors in surgery | Healy, N. A., et al. (2012). "Role models and mentors in surgery." The American Journal of Surgery 204(2): 256-261. | Systematic review | A comprehensive PubMed search of the literature on the subject of role models and mentors was performed using the following keywords: “Mentors,” “Mentorship,” and “Role Models” alone and in conjunction with the words “medicine” and “surgery.” | A number of studies have highlighted the importance of positive role models and mentors in influencing medical students’ ultimate career decisions. This article sought to review the relevant literature in relation to mentors and role models in surgery. | Surgery | 1. Significant positive correlation between the presence of a mentor and a desire to pursue a career in academic medicine. 2. Trainees who had a mentor were more likely to pursue research and be more productive in terms of research output and grant attainment. 3. Research fellows in receipt of mentorship were more likely to act as mentors to others. 4. There is minimal research into the impact of mentoring programs on student interest in surgery or junior doctor satisfaction and committal to a career in surgery. 5. Research pertaining to the importance of mentorship among surgical trainees is minimal. Whether the lack of mentors has a role in the attrition rate of surgical trainees is unclear. 6. To maximize work-life balance, it is important that surgeons, trainees, and students develop “life mentors” to deal with the demands of pursuing a busy career path while maintaining a satisfactory personal life. These mentors are not necessarily required to have a medical background but should be available to offer advice and support regarding dealing with life in general. 7. The surgical team can have a strong influence on medical students, with individual members acting as positive or negative role models or mentors to the students. 8. Women currently hold a minority of the high-ranking surgical positions, leading to a lack of well-developed female surgical networks. As a result, women in surgery are required to seek mentorship from male mentors. This may generate difficulty because many male senior faculty members assume that women will not progress in surgery and therefore direct their attention elsewhere. 9. Surgeons need to become more aware of the example that they set for junior colleagues. To encourage young people to embark on and persist with a surgical career, surgery must provide appropriate role models as well as formal mentorship programs. | 1. Senior mentor programs supported by peer mentoring structures would be beneficial. There is also great potential for online mentoring programs with the possible incorporation of social networking facilities and web-conferencing tools for geographically distributed mentor-mentee relationships. |
| 37 | Social media in the mentorship and networking of physicians: Important role for women in surgical specialties | Luc, J. G. Y., et al. (2018). "Social media in the mentorship and networking of physicians: Important role for women in surgical specialties." The American Journal of Surgery 215(4): 752-760. | Cross-sectional study (qualitative study) | A 35-item survey investigating trainee and physician social media use was designed using online survey software and distributed via social media and email announcements with an introductory letter explaining the purposes of the survey. Inclusion required respondents to be practicing physicians or trainees pre- paring for careers as physicians (including fellows, residents, medical students, and pre-med students). All responses were voluntary and anonymous. | Social media may be a useful supplement to physician and trainee interactions; however, its role in enhancing mentorship has not been described. | Practicing physicians or trainees preparing for careers as physicians (including fellows, residents, medical students, and pre-med students). | 1. Women in surgical specialties were more likely to describe the specialty as being dominated by the opposite sex and to be mentored by the opposite sex though wish to be mentored by individuals of the same sex. 2. Respondents in surgical specialties were also more likely to report using social media to build a network of same-sex mentorship. 3. Social media serves as a valuable tool to enhance the networking and mentorship of surgeons, particularly for women in surgical specialties who may lack exposure to same-sex mentors at their own institution.  4. Social media serves as an informal platform for communication and serendipitous relationships to occur based on mutual interests and goals. 5. Beyond individual mentorship, social media also has the potential to establish a community of mentors for mentees with expertise to aid multiple areas or stages of their career and to provide the opportunities and experiences to reach their full potential. | 1. Longitudinal studies surrounding the effectiveness of this emerging method of mentorship (using social media) are warranted. |
| 38 | Strategies for building an effective mentoring relationship | Sanfey, H., et al. (2013). "Strategies for building an effective mentoring relationship." The American Journal of Surgery 206(5): 714-718. | Editorial | Not explicitly stated though it would appear that this is an editorial built upon a literature review | Mentoring has been recognized as a critical aspect of the professional and/or personal development of the student, resident or faculty member. This career development resource discusses strategies for building effective mentoring relationships and outlines some of the challenges to contemporary mentoring. | Surgery: focused on women surgeons and residents | 1. A mentoring relationship may begin serendipitously from a common interest or goal. One partner will generally take the initiative to formalize the relationship. At the onset, the mentee needs to have clear goals. In seeking mentors, medical students should familiarize themselves with the faculty members (and residents) at their institution by researching departmental Web sites and talking to other students. 2. The mentoring relationship is one of collaboration that should ultimately benefit both parties.  3. Challenges may arise because of the intensity of mentoring relationships and the potential for misunderstandings, such as cross-race and/or cross-gender relationships in which mentors may have trouble identifying with their mentees and vice versa, and also generational differences. | 1. Mentors should recognize when they are unable to resolve certain problems (eg mentee develops clinical depression or experience substance abuse) and should refer challenged mentees to their primary care physicians or suggest consultation with specialists such as study counselors or psychologists. |
| 39 | Surgical Education and the Mentor–Student Relationship | Konstantakos, A. K. (2003). "Surgical education and the mentor-student relationship." Curr Surg 60(5): 547-548. | Opinion | Not applicable. | Not stated. | Surgery | 1. Education is a 2-way street. To teach is to learn and to learn is to teach. This applies to the attending-resident/student relationship. | 1. Both student and teacher should be responsive to open questioning. Attendings can stimulate thinking in students and residents by asking progressively probing questions. Attendings should be willing to learn from the residents too.The attitude that an attending is an “old dog that can’t learn new tricks” stifles education. 2. A major barrier to attending–resident communication is personality conflicts. The attending should point out the strengths and weaknesses of the resident in a positive manner. The key is communication. 3. Attendings should understand what the residents' goals are. 4. Timing of feedback -- Attendings should give personal verbal feedback and constructive criticism early-on in the rotation to establish a positive rapport and well-defined working relationship with the house-staff. |
| 40 | Surgical education: Lessons from parenthood | Burlew, C. C. (2017). "Surgical education: Lessons from parenthood." The American Journal of Surgery 214(6): 983-992. | Editorial | Not stated. | Surgery and parenting are similar in certain aspects. Educators and parents relied on modelling the behavior of others, or trial and error techniques. Mentorship and role models have played a critical role in professional development and continue to have a profound impact. | Surgery | 1. Mentorship has a large impact on career choices and specializations. | 1. Having a single mentor is not truly realistic, but rather having a web or network of mentors was far more effective. 2. Promote autonomy and navigate failure. 3. Cultivate grit. 4. Provide feedback. |
| 41 | Surgical Mentors and Role Models: Prevalence, Importance and Associated Traits | Healy, N. A., et al. (2012). "Surgical Mentors and Role Models: Prevalence, Importance and Associated Traits." Journal of Surgical Education 69(5): 633-637. | Cross-sectional study (qualitative study) | A questionnaire was distributed to senior undergraduate medical students in 1 medical school, and postgraduate surgical trainees (members of the Association of Surgeons in Training (ASIT) in the UK and Ireland. The survey included questions about the availability of mentors and role models and explored mentorship process. A total of 163 medical students and 216 surgical trainees completed the questionnaire. | Role models and mentors play an impor- tant part in attracting undergraduates into various medical spe- cialties. However, little is known about the part played by role models and mentors in the context of surgery. The aim of this survey was to elucidate medical students’ and surgical trainees’ experiences of role models and to determine how mentoring works in practice. We also set out to identify traits associated with successful role models and mentors. | Surgery | 1. A very small proportion of medical students and trainees claim to have a surgical mentor or a role model. 2. Mentorship for both Irish students and UK trainees was largely informal, ad hoc, with individual selection of mentors. Showing a lack of formal mentoring programs among students and surgical trainees in the UK and Ireland. 3. Traits identified by both medical students and surgical trainees should act as a guide for surgeons. Under clinical competence traits, such as good clinical knowledge, effective interpersonal skills, and enthusiasm for surgical practice are considered important. On personal qualities, trainees consider leadership, integrity, objectivity, and professionalism to be essential traits, while students rate attitudes displayed towards students and residents, compassionate and caring, and also leadership to be important traits for surgical mentors. For mentors to be considered good teachers, they must have good communication skills and possess an ability to make difficult topics understood. Finally, to be classed as a good research mentor, surgeons should be able to promote interest in research, involve junior members in research, and also have a background in research. | 1. To encourage medical students to pursue a surgical career, surgery must provide appropriate positive role models, in addition to formal mentorship programs. |
| 42 | Surgical residents as medical student mentors | Nguyen, S. Q. and C. M. Divino (2007). "Surgical residents as medical student mentors." The American Journal of Surgery 193(1): 90-93. | Cross-sectional study (qualitative study) | In 2004–2005, 117 medical students at Mount Sinai School of Medicine completed their third-year surgery clerkship. They were asked to complete an anonymous survey regarding a career in surgery and surgical mentors. A total of 107 students (91%) completed the survey. | Medical students’ decreasing interest in surgery may be caused by the inadequate availability of role models. We believe that surgical residents show the qualities of outstanding surgical mentors and are in a key position to influence students’ career choices. This study explored students’ views regarding surgical mentors, specifically examining their association of mentoring qualities with their resident teachers. | Surgery | 1. The surgery clerkship positively influenced students’ perceptions of surgical careers, likely due to identifying appropriate mentors. 2. Both faculty and residents can contribute significantly in the mentoring process. 3. Residents outscored attendings in 12 of the 14 qualities noted as important for a clinical mentor. A large contribut- ing factor to this is the amount of time spent together. Students may score residents higher simply because their interactions are much more extensive than with attendings. 4. Clinical competence and fund of knowledge were the 2 attributes for which students believed attending physicians fared better than residents. | 1. Small sample size. This was a survey of one class of students at one medical school. A broader examination of multiple classes of students at multiple medical schools may minimize selection bias. 2. This study did not specifically examine the negative factors that may have had a significant impact on students’ identification of mentors. 3. Formal mentoring relationships can be set up between certain medical student and senior resident pairs. Multiple pairings can be overseen further by a single attending surgeon. 4. To increase resident participation in mentoring, 1-year teaching fellowships can be set up as alternatives to the laboratory years that some residents elect to undertake. |
| 43 | The advisory dean program: a personalized approach to academic and career advising for medical students | Macaulay, W., et al. (2007). "The advisory dean program: a personalized approach to academic and career advising for medical students." Acad Med 82(7): 718-722. | Observational study | Advisory deans and the dean for student affairs, familiar with resources for academic development, student support, and extracurricular activities, operate at the nexus of the program, providing personalized mentoring and advising for each student. | Catalyzed by negative student feedback regarding career advising and a perceived disconnection between faculty and students, the AD program was set up to enhance students' professional development throughout their student training. | Medical students | 1. Out of 152 students, 104 (68%) provided feedback, with 93 (89%) of the respondents reporting the AD Program as a valuable initiative.  2. On the basis of our experience so far, we expect that this organized program of advising will make the transition from student to responsible, compassionate, and competent physician an easier, more enjoyable, enlightening process. | None |
| 44 | The core of mentorship: medical students' experiences of one-to-one mentoring in a clinical environment | Kalen, S., et al. (2012). "The core of mentorship: medical students' experiences of one-to-one mentoring in a clinical environment." Adv Health Sci Educ Theory Pract 17(3): 389-401. | Observational study | A mentoring program was set up where all medical students were offered a mentor during their first clinical courses; years 3–4. The mentors were physicians and their role as mentors was to support the students and act as sounding-boards, not to teach or assess knowledge. A qualitative approach with individual interviews (N = 12) and inductive content analysis was chosen to investigate and interpret the meaning of mentorship. | This study aimed to get a deeper understanding of the meaning of mentorship seen from the perspective of undergraduate medical students. | Third to fourth year medical students | 1. The results comprise three overarching themes: Space, Belief in the future and Transition. Having a mentor gave a sense of security and constituted a ‘free zone’ alongside the undergraduate programme. 2. We would argue that one-to-one mentoring can create conditions for medical students to start to develop some parts of the professional competences that are more elusive in medical education programmes, such as reflective capacity, emotional competence and the feeling of belonging to a community. | 1. It might be relevant to include one-to-one mentoring programmes in undergraduate medical education. 2. Further studies are needed to clarify the extent to which one-to-one mentorship can be linked to these areas of professional development that have received increased attention in the medical profession in recent years. 3. Since one-to-one mentoring involving a large number of students requires extensive resources, more knowl- edge is also needed about medical students’ experiences of other forms of mentoring. |
| 45 | The Legacy of Teaching Medical Professionalism for Promoting Professional Practice: A Systematic Review | Guraya, S., et al. (2016). "The Legacy of Teaching Medical Professionalism for Promoting Professional Practice: A Systematic Review." Biomedical and Pharmacology Journal 9: 809-817. | Systematic review | In May 2016, the databases of ISI web of knowledge, Scopus, the Cochrane Library, and MEDLINE were searched for the full-text English-language articles published during 2005 through 2015 using the keywords “Medical professionalism” OR “Role modelling” OR “Teaching and learning” OR “Mentoring” OR “Hidden curriculum” OR “Reflective practice”. This search showed 974 articles and further shortlisting and exclusion of non-relevant material selected 48 articles for detailed analysis in this systematic review. | Teaching the culture-specific, context-based and societal characteristics of medical professional are challenging. This work identifies the teaching strategies for medical professionalism drawn from the published articles that have validated their effectiveness through some objective measures. | Medical students | 1. The most powerful and effective strategies with profound impact has been imparted by role modelling, mentoring, hidden curriculum, reflective practice, and by effective communication. 2. Medical faculty can exert positive and exemplary role by practicing with integrity, respect for patients, and altruism. 3. The professional conduct of medical faculty by role modelling in hidden curricula and by teaching core principles of reflective practice and mentoring can potentially encourage and enhance a professionally rich culture among medical students. | 1. Academics are urged to collaborate worldwide to share ideas in developing excellence in role modelling and reflective that will, in turn, establish high standards of patient care and professional excellence. 2. Future research is needed to explore the association between culture, versatility and gender while recruiting physician role models and mentors. |
| 46 | The mentoring needs of trainees in family practice | Belle Brown, J., et al. (2012). "The mentoring needs of trainees in family practice." Education for Primary Care 23(3): 196-203. | Cross-sectional study (qualitative study) | Eight focus groups and 16 individual interviews were used to collect data from 49 medical students and 29 family practice residents. Interviews and focus groups were audio- taped and transcribed verbatim. The analysis was iterative and interpretive, using both individual and team analyses. | This study attempts to address these gaps in the extant literature by determining the essential components of a mentoring relationship which would benefit medical students and family practice residents. This qualitative study examined medical students' and family practice residents' ideas, perceptions, and experiences of being mentored and their expectations of the mentoring experience. | Medical students and family practice residents | 1. Early and continuous exposure to family practice mentors is important in career development. 2. Specific learning needs were identified at the practice, system, and personal level. 3. Viewing these various components of the mentoring relationship as a whole illuminates the evolutionary nature and fluidity of the mentoring experience. | 1. The authors strongly endorse the concept of mentoring medical students and family practice residents during their training. |
| 47 | The Munich-Evaluation-of-Mentoring-Questionnaire (MEMeQ) – a novel instrument for evaluating protégés’ satisfaction with mentoring relationships in medical education | Schäfer, M., et al. (2015). "The Munich-Evaluation-of-Mentoring-Questionnaire (MEMeQ) – a novel instrument for evaluating protégés’ satisfaction with mentoring relationships in medical education." BMC medical education 15(1): 201. | Cross-sectional study (qualitative study) | Based on two existing questionnaires, the authors developed an instrument to evaluate the weighted satisfaction of mentoring relationships, emphasizing the protégés' individual expectations and needs. Protégés first define individual areas of interest in their mentoring relationship, then assign relative levels of personal importance to them and finally rate their individual level of satisfaction with their mentors' support in each area of interest. In order to evaluate psychometric properties as well as acceptance and feasibility the investigators conducted a multi- method-study. | Despite the widespread recognition of the importance of mentoring in medical education, valid and reliable instruments for evaluating the relationship of mentors and protégés are lacking. The aim of this study was to develop a feasible instrument to measure the satisfaction with mentoring relationships. | Medical students | 1. The evaluation of mentoring relationships in medical education is important.  2. Satisfaction seems to be the most reliable predictor for the success of mentoring rela- tionships. 3. It is necessary to consider protégés expectations and needs.  4. MEMeQ is a reliable, valid and flexible instrument for measuring the weighted satisfaction of protégés with their individual mentoring relationship in medical education. | None |
| 48 | The role of the teacher in remediating at-risk medical students | Winston, K. A., et al. (2012). "The role of the teacher in remediating at-risk medical students." Medical Teacher 34(11): e732-e742. | Cross-sectional study (qualitative study) | Extensive qualitative data from student surveys and in-depth teacher interviews, along with quantitative student performance data, produced a rich description of remediation processes. | Previous work identified complex ingredients of a remediation programme for at-risk medical student. The paper explores the teachers’ role in this intervention, aiming to expand and deepen understanding of remediation methods in medical education. | At-risk medical students | 1. Remediation should support emotional needs and foster cognitive and metacognitive skills for self-regulation and critical thinking. 2. This community should foster curiosity and joy for learning, using collaborative exploratory dialogue that revolves around their experiences of failure, success and deep engagement with the subject matter of their courses. 3. Teachers of remediation can mediate these processes through embodiment of five core roles; facilitator, nurturing mentor, disciplinarian, diagnostician and modeller of desired skills, attitudes and behaviours. | 1. This practical theory draws together elements of teaching excellence supported by a wide range of literature to create a unique blend tailored to the high cognitive and affective demands of remediation in medical education, and promises value in remediation in other higher educational contexts. |
| 49 | What makes a good clinical student and teacher? An exploratory study | Goldie, J., et al. (2015). "What makes a good clinical student and teacher? An exploratory study." BMC medical education 15: 40. | Cross-sectional study (qualitative study) | A qualitative approach using individual interviews with educational supervisors and focus groups with senior clinical students was used. Data was analysed using a “framework” technique. | What makes a good clinical student is an area that has received little coverage in the literature and much of the available literature is based on essays and surveys. This paper aims to explore this topic as well as gain greater insight as to what makes a good clinical teacher. | Clinical students | 1. Good clinical students are proactive in their learning; an important quality where students are expected to be active in managing their own learning. Good clinical students share similar characteristics with good clinical teachers. 2. A teacher’s enthusiasm and non-cognitive abilities are as important as their cognitive abilities. 3. Student learning in clinical settings is a collective responsibility. | 1. Our findings could be used in tutor training and for formative assessment of both clinical students and teachers. This may promote early recognition and intervention when problems arise. |
| 50 | What makes them different? An exploration of mentoring for female faculty, residents, and medical students pursuing a career in surgery | Bettis, J., et al. (2019). "What makes them different? An exploration of mentoring for female faculty, residents, and medical students pursuing a career in surgery." The American Journal of Surgery 218(4): 767-771. | Cross-sectional study (qualitative study) | Semi-structured interviews were held with a purposive sample of 24 female surgical faculty, residents, and aspiring medical students from one institution between November 2018 and January 2019. Interview transcripts were analyzed using traditional thematic analysis methods aided by computerized software. | This qualitative study examines the roles of mentoring and gender in choosing and continuing in a surgical career for women across the continuum. | Residents and aspiring medical students - surgery | 1. The use of a mosaic approach in seeking mentoring to match one's personal and career-relevant support needs was described frequently.  2. Same-gender role models were more important for early career women, while leadership mentoring and coaching were more desired by later career women. 3. Gender differences in mentoring were identified but some of these differences may apply equally to women and men. 4. This study found that seeing a same-gender role model was influential for early-career women surgeons. | 1. There is a need for both ongoing mentoring as well as multiple types of mentors as needs change throughout one's career. In addition, though same-gender mentoring differ- ences were frequently noted, these trends may apply equally to women and men. |
| 51 | Why do women choose or reject careers in academic medicine? A narrative review of empirical evidence | Edmunds, L. D., et al. (2016). "Why do women choose or reject careers in academic medicine? A narrative review of empirical evidence." The Lancet 388(10062): 2948-2958. | Systematic review | Following a systematic review, two reviewers, working independently, selected studies that reported results by gender for reasons associated with medical students or resident doctors choosing or rejecting careers in academic medicine. Methodological quality was assessed using the relevant elements of the Critical Appraisal Skills Programme tools. | Women are under-represented in academic medicine. We reviewed the empirical evidence focusing on the reasons for women’s choice or rejection of careers in academic medicine | Female medical students and residents | 1. There was consistent evidence for four of these themes: women are interested in teaching more than in research; participation in research can encourage women into academic medicine; women lack adequate mentors and role models; and women experience gender discrimination and bias. 2. The evidence was conflicting on four themes: women are less interested in research than men; women lose commitment to research as their education and training progress; women are deterred from academic careers by financial considerations; and women are deterred by concerns about work–life balance. | 1. Inconsistency of findings across studies suggests significant opportunities to overcome barriers by providing a more enabling environment. |
